# Incidence, Mortality and Survival Time Trends of Brain and CNS Tumours in the Canton of Zurich (Switzerland) Between 1980 and 2021

**DOI:** 10.1002/cam4.71052

**Published:** 2025-07-21

**Authors:** Miriam Wanner, Flurina Suter, Manuela Limam, Dimitri Korol, Sabine Rohrmann

**Affiliations:** ^1^ Cancer Registry Zurich, Zug, Schaffhausen and Schwyz Institute of Pathology and Molecular Pathology, University Hospital Zurich Zurich Switzerland; ^2^ Epidemiology, Biostatistics and Prevention Institute University of Zurich Zurich Switzerland

**Keywords:** brain and CNS tumours, cancer registry, incidence, mortality, relative survival, Switzerland, time trends

## Abstract

**Purpose:**

We aimed to analyse trends in incidence, mortality and 5‐year relative survival of malignant and benign/borderline brain and central nervous system (CNS) tumours between 1980 and 2021 in the Canton of Zurich, Switzerland, stratified by sex, age group, behaviour and histological subtypes.

**Methods:**

We used incidence data from the Cancer Registry of Zurich, Zug, Schaffhausen and Schwyz, including primary benign/borderline and malignant tumours diagnosed between 1980 and 2021 in the Canton of Zurich in patients aged ≥ 15 years (*N* = 10,226). Mortality data were provided by the Swiss Federal Statistical Office (*N* = 3514). We calculated age‐standardised incidence and mortality rates per 100,000 person‐years and used Joinpoint to analyse trends.

**Results:**

The age‐standardised incidence rate of malignant tumours was stable over time (around 7.7–8.2 per 100,000 person‐years in men and 4.6–5.2 in women), while the rate of benign/borderline tumours increased from 3.8 in 1980–1990 to 10.8 in 2011–2021 in men and from 5.7 to 19.1 in women. The age‐standardised mortality rate remained stable over time for malignant tumours (around 5.5–6.1 in men and 3.5–4.0 in women) but significantly decreased for benign/borderline tumours (from 1.0 to 0.5 in men and from 1.2 to 0.5 in women). Age‐standardised 5‐year relative survival increased from around 80% in 1980–1989 up to > 90% in 2011–2017 for benign/borderline tumours and from < 20% to around 30% for malignant tumours. There was a small survival advantage in women compared to men.

**Conclusions:**

We observed an increase in incidence and a decrease in mortality rates for benign/borderline tumours, while both rates remained stable for malignant tumours. Five‐year relative survival improved over time. The increasing incidence rates in benign/borderline tumours may be due to improved diagnostic techniques and an increasing use of CT scans, as reported in other countries. The increase in relative survival may reflect earlier detection and better treatment options.

## 1 Introduction

According to the Swiss Cancer Report [1], malignant brain and central nervous system (CNS) tumours accounted on average for 1.7% (men) and 1.4% (women) of all newly diagnosed cancer cases in Switzerland in the years 2013–2017. At the same time, these tumours were responsible for 3.3% (men) and 2.7% (women) of all cancer deaths. The standardised incidence rates for this time period were 7.8 per 100,000 person‐years in men and 5.1 in women [1]. Five‐year relative survival was 26% in men and 29% in women in 2013–2017. According to the report, both incidence and mortality rates in men and women remained stable between 1988 and 2017 [1]. A Swiss study including both benign/borderline and malignant brain and CNS tumours for the period 2010–2014 reported standardised incidence rates of 8.4 (men) and 5.1 (women) per 100,000 person‐years for malignant tumours and 6.6 (men) and 12.6 (women) for benign/borderline tumours [2]. While for malignant tumours, the rate for men was about 1.5 times higher compared with the rate for women, the opposite was observed for benign/borderline tumours with rates for women that were almost double the rates for men.

Global rates of brain and CNS tumours vary greatly [3, 4, 5], with mostly higher rates in Europe and the US and lower rates in Africa and Asia. Furthermore, rates are generally higher in high‐income countries compared with low‐ and middle‐income countries [4], which has also been shown in a comparison between Georgia and Switzerland [6]. However, many studies only included malignant brain and CNS tumours [3, 4, 5], for example, due to a lack of available data regarding benign/borderline tumours in cancer registries around the world.

Large variations across the world were also observed for survival of brain tumours [7]. Age‐standardised 5‐year net survival was between 20% and 40% in 2010–2014 for most countries and was lowest in Thailand (14.7%) and highest in Croatia (42.2%) [8].

In the cancer registry Zurich, Zug, Schaffhausen and Schwyz in Switzerland, data on both malignant and benign/borderline brain and CNS tumours have been registered for the largest Swiss Canton (Zurich) since 1980. This gave us the opportunity to investigate trends regarding incidence, mortality and survival over a long period of time, including both malignant and benign/borderline tumours in a high‐income country with a very good health system.

The aim of this study was to analyse trends in incidence, mortality and 5‐year relative survival of malignant and benign/borderline brain and CNS tumours between 1980 and 2021 with follow‐up dates up until 31.12.2022 in the Canton of Zurich, Switzerland. Analyses were stratified by sex, age group, behaviour and histological subtypes.

## 2 Methods

### 2.1 Data Sources and Inclusion Criteria

The population‐based cancer registry of the canton of Zurich, which covers a population of 1.56 million inhabitants in 2021, started to register data in 1980. Cancer registration for the canton of Zug started in 2011 and for the cantons of Schaffhausen and Schwyz in 2020, resulting in the now called cancer registry Zurich, Zug, Schaffhausen and Schwyz. Due to the later start of cancer registration in the latter three cantons, we only included data of the canton of Zurich in the present analyses. Cancer patients need to have their main place of residence in the canton of Zurich to be registered in our database.

We included all primary tumours with topography codes C70‐C72 as well as C75.1, C75.2 and C75.3, including benign, borderline and malignant cases, diagnosed between 1980 and 2021 in the Canton of Zurich, Switzerland, in patients aged 15 years and older at diagnosis in order to be compliant with other studies [4, 8]. The tumours were classified into subgroups based on histology codes using the definitions in the latest CBTRUS Statistical Report [9]. The definition of the subgroups is displayed in Table S1. The behaviour code 3 was used to categorise malignant tumours, while the behaviour codes 0 (benign) and 1 (unclear whether benign or malignant) were used to categorise benign/borderline tumours. Table S1 also displays the histopathological codes in combination with the behaviour codes. The distribution of the included tumours according to broader histology subgroups (used for stratification in the analyses) and behaviour is displayed in Table S2.

The dataset included information regarding the date of diagnosis, the age at diagnosis, topography and morphology according to ICDO‐3 (International Classification of Diseases in Oncology), basis of diagnosis (indication of death certificate only (DCO) cases), vital status at follow‐up and date of follow‐up, which was available at least up to 31.12.2022 for most patients. In the case of date of follow‐up earlier than that, the vital status was set to lost to follow‐up using the date of last contact as follow‐up date.

In general, the data quality in the cancer registry Zurich, Zug, Schaffhausen and Schwyz is good [10]. According to data from 1997 to 2014, the percentage of DCO cases was 2.6% and the percentage of morphologically verified cases was 93.3% [10].

The mortality data were provided by the Swiss Federal Statistical Office and were based on the national cause of death statistics. We used data from 1980 to 2021. The main cause of death was coded according to ICD8 (8th revision of the International Classification of Disease) up to 1994 and according to ICD10 from 1995 onwards. For the mortality analyses, we used the ICD8 codes 191 and 192 for malignant tumours, 225 for benign tumours, and 238 for tumours of unknown behaviour, and the ICD10 codes C70–C72, C75.1, C75.2, C75.3, D32–D33, D42–D43, D35.2, D35.3, D35.4, D44.3, D44.4, D44.5. For the survival analyses, we excluded DCO cases.

### 2.2 Statistical Analyses

We calculated age‐standardised incidence and mortality rates per 100,000 person‐years using the 1976 European standard population [11] and mid‐year population estimates. The analyses were stratified by sex, age group and behaviour. In additional analyses, we also stratified by histological subtypes. Behaviour was summarised as benign/borderline (behaviour = 0, 1) versus malignant (behaviour = 3). For the age‐stratified analyses, the following age groups were used: 15 to < 35 years, 35 to < 55 years, 55 to < 75 years and ≥ 75 years.

We estimated age‐standardised 5‐year relative survival as the ratio of the observed survival to the expected survival without taking cause of death into account. Expected survival was calculated based on the Ederer II method [12] and applied to all‐cause mortality tables for the canton of Zurich supplied by the Federal Statistical Office. Death probabilities based on age‐, sex‐ and calendar year‐specific death rates were interpolated and smoothed using the Elandt Johnson formula [13]. Relative survival was developed as an estimator of net survival, which is commonly used when estimating patient survival using data from population‐based cancer registries [14]. Relative survival was estimated using the strs command in Stata Statistical Software (StataCorp LP, version 16). Cohort analyses were used to derive relative survival estimates for all time periods (2010–2017 for the latest period in order to provide at least 5 years of follow‐up).

We used STATA/SE Version 16 to calculate incidence, mortality and relative survival rates (Stata Corporation, College Station, TX, USA). Incidence and mortality time trends were assessed using Joinpoint (Joinpoint Trend Analysis Software Version 5.2.0., June 2024; US National Cancer Institute, Division of Cancer Control and Population Sciences, Surveillance Research Program). The Joinpoint software fits models starting with a minimum number of joinpoints (commonly 0 joinpoints, which corresponds to a straight line) and tests whether more joinpoints result in a significantly better model fit and should be added to the model in order to adequately assess changes over time. The weighted Bayesian Information Criterion method was used for model selection. We defined the maximum number of joinpoints as five based on the recommendations in the Joinpoint Manual. Furthermore, we set the minimum number of observations from a joinpoint to either end of the data as two, and the minimum number of observations between two joinpoints as two (including any joinpoint that falls on an observation). The dependent variables were the age‐standardised incidence and mortality rates; the independent variable was the calendar year. Age group and behaviour were defined as by‐variables. The models provide annual percentage changes (APC) for each identified trend between two joinpoints and the average annual percentage change (AAPC) as a summary measure over the whole period of observations (1980–2021). Log‐transformation of the dependent variable was used because of the non‐normality of the data. The AAPC are presented with 95% confidence intervals (95% CI).

## 3 Results

In total, 10,226 incident brain and CNS tumours were registered between 1980 and 2021 in the canton of Zurich, Switzerland, of which 3971 (38.8%) were classified as malignant and 6255 (61.2%) as benign/borderline. In the same period, 3514 deaths due to brain and CNS tumours were registered, of which 2982 (84.9%) were classified as malignant and 532 (15.1%) as benign/borderline. Tables 1 and 2 display the number of cases by sex, behaviour, period and age group for incidence and mortality, respectively.

**TABLE 1:** Absolute number (% men versus women) of incident brain and CNS tumours by sex, behaviour, diagnosis period and age group, 1980–2021, canton of Zurich, Switzerland.

	All tumours		Benign/borderline tumours		Malignant tumours	
	Men	Women	Men	Women	Men	Women
Overall	4418 (43.2)	5808 (56.8)	2126 (34.0)	4129 (66.0)	2292 (57.7)	1679 (42.3)
Diagnosis period						
1980–1990	699 (46.2)	813 (53.8)	228 (34.2)	439 (65.8)	471 (55.7)	374 (44.3)
1991–2000	823 (46.5)	946 (53.5)	316 (33.9)	617 (66.1)	507 (60.6)	329 (39.4)
2001–2010	1108 (42.6)	1490 (57.4)	537 (33.5)	1068 (66.5)	571 (57.5)	422 (42.5)
2011–2021	1788 (41.1)	2559 (58.9)	1045 (34.3)	2005 (65.7)	743 (57.3)	554 (42.7)
Age group						
15 to < 35 years	422 (49.1)	438 (50.9)	195 (42.0)	269 (58.0)	227 (57.3)	169 (42.7)
35 to < 55 years	1193 (44.6)	1483 (55.4)	595 (34.9)	1111 (65.1)	598 (61.6)	372 (38.4)
55 to < 75 years	1959 (45.1)	2385 (54.9)	884 (35.3)	1620 (64.7)	1075 (58.4)	765 (41.6)
≥ 75 years	844 (36.0)	1502 (64.0)	452 (28.6)	1129 (71.4)	392 (51.2)	373 (48.8)

**TABLE 2:** Absolute number (% men versus women) of deaths due to brain and CNS tumours by sex, behaviour, period and age group, 1980–2021, canton of Zurich, Switzerland.

	All tumours		Benign/borderline tumours		Malignant tumours	
	Men	Women	Men	Women	Men	Women
Overall	1887 (53.7)	1627 (46.3)	197 (37.0)	335 (63.0)	1690 (56.7)	1292 (43.3)
Period						
1980–1990	400 (49.5)	408 (50.5)	57 (33.9)	111 (66.1)	343 (53.6)	297 (46.4)
1991–2000	413 (55.6)	330 (44.4)	37 (38.9)	58 (61.1)	376 (58.0)	272 (42.0)
2001–2010	446 (53.7)	384 (46.3)	49 (37.7)	81 (62.3)	397 (56.7)	303 (43.3)
2011–2021	628 (55.4)	505 (44.6)	54 (38.8)	85 (61.2)	574 (57.7)	420 (42.3)
Age group						
15 to < 35 years	74 (53.6)	64 (46.4)	3 (37.5)	5 (62.5)	71 (54.6)	59 (45.4)
35 to < 55 years	387 (59.7)	261 (40.3)	14 (37.8)	23 (62.2)	373 (61.0)	238 (39.0)
55 to < 75 years	937 (55.5)	750 (44.5)	72 (44.4)	90 (55.6)	865 (56.7)	660 (43.3)
≥ 75 years	489 (47.0)	552 (53.0)	108 (33.2)	217 (66.8)	381 (53.2)	335 (46.8)

### 3.1 Incidence

In 1980–1990, the overall age‐standardised incidence rate of malignant and benign/borderline brain and CNS tumours was 11.5 per 100,000 person‐years in men and 10.8 in women. In 2011–2021, the respective rates were 18.5 in men and 24.1 in women. Over the whole period (1980–2021), there was an AAPC of 1.2 (95% CI 0.5, 2.1) for men and 1.8 (1.1, 2.3) for women. Table 3 displays the age‐standardised incidence rates by sex, behaviour and period.

**TABLE 3:** Age‐standardised incidence rates of brain and CNS tumours (per 100,000 person‐years) by sex, behaviour and period, 1980–2021, canton of Zurich, Switzerland.

	Malignant tumours		Benign/borderline tumours	
	Men	Women	Men	Women
Period				
1980–1990	7.7	5.1	3.8	5.7
1991–2000	8.2	4.6	5.1	7.9
2001–2010	8.1	5.2	7.4	12.6
2011–2021	7.7	5.0	10.8	19.1

**FIGURE 1:**
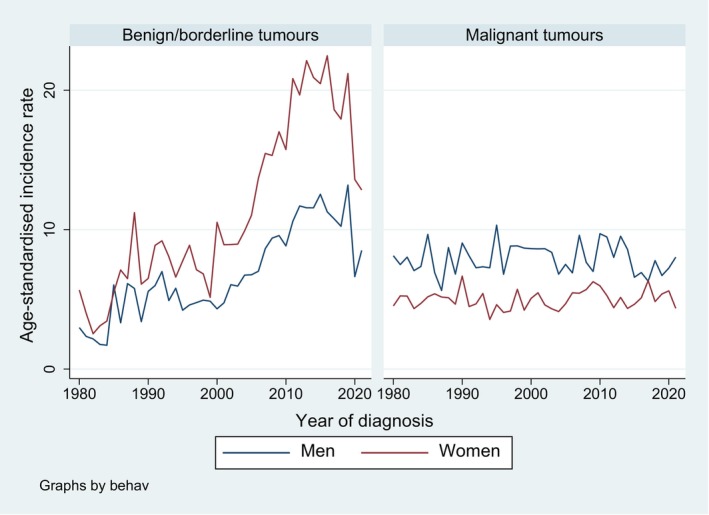
Trends in age‐standardised incidence rates for brain and CNS tumours (per 100,000 person‐years) from 1980 to 2021, by sex and behaviour, canton of Zurich, Switzerland.

Age‐standardised incidence rates were higher in women than in men for benign/borderline brain and CNS tumours, while they were higher in men than in women for malignant brain and CNS tumours (Figure 1). While the incidence rate increased over time for benign/borderline tumours (AAPC of 2.1 [1.1, 4.8] for men and of 3.0 [2.1, 4.0] for women), it remained relatively stable for malignant tumours (AAPC of −0.1 [−0.5, 0.4] for men and of 0.2 [−0.2, 0.6] for women).

For benign/borderline tumours, there was one joinpoint in men with significantly increasing APC up to 2019 and a nonsignificant decrease thereafter; and one in women in 2016 with significantly increasing APC (4.8 [4.2, 5.9]) up to 2016 and significantly decreasing APC (−8.8 [−25.1, −1.1]) thereafter. No joinpoints were estimated in the models for malignant tumours.

**FIGURE 2:**
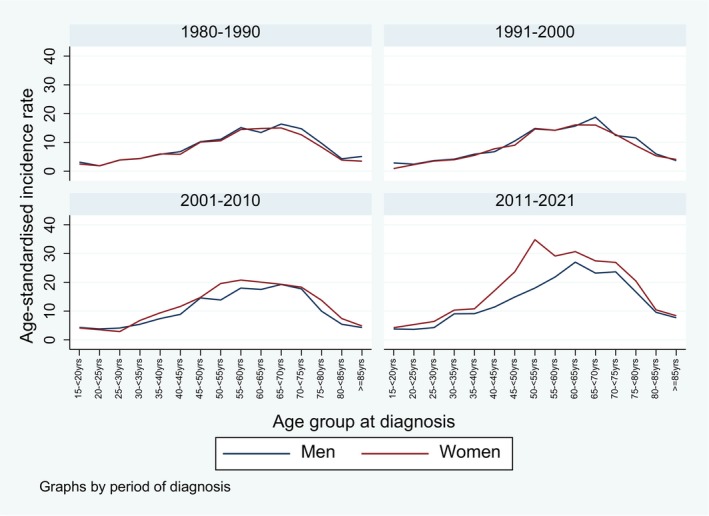
Age‐standardised incidence rates for brain and CNS tumours (per 100,000 person‐years) from 1980 to 2021, by sex, period and age group, canton of Zurich, Switzerland.

**FIGURE 3:**
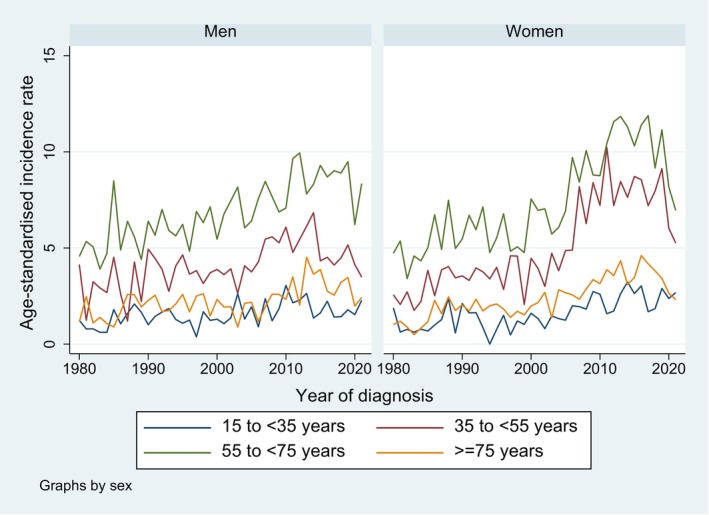
Trends in age‐standardised incidence rates for brain and CNS tumours (per 100,000 person‐years) from 1980 to 2021, by sex and age group, canton of Zurich, Switzerland.

Figure 2 shows the distribution across age groups, by period and sex. Highest incidence rates were observed between about 45 and 75 years of age for both men and women. While the distribution was similar for men and women in the first period (1980–1990), women had slightly higher rates, especially in the middle ages, in later periods. Furthermore, rates were generally higher in the middle age groups in later periods compared to earlier periods. This is supported by Figure 3 showing that the increase over time was more pronounced in the middle age categories (35–54 and 55–74 years), especially in women. Independent of time, the highest incidence rates were observed in the 55–74‐year‐old patients, both in men and women. We found significantly increasing AAPC for all age groups and both sexes except for the 35 to < 55 year old men where the increase was only significant up to 2014 followed by a nonsignificant decrease.

**FIGURE 4:**
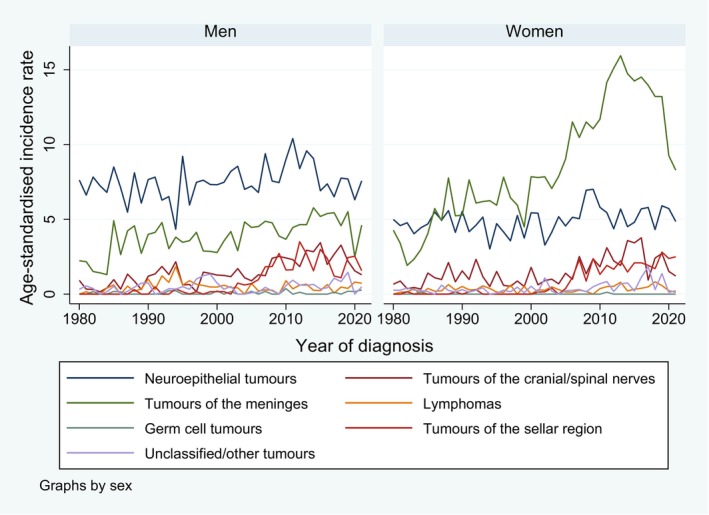
Trends in age‐standardised incidence rates for brain and CNS tumours (per 100,000 person‐years) from 1980 to 2021, by sex and histology group, canton of Zurich, Switzerland.

Table S2 displays the distribution of the histopathological subgroups used in this study by behaviour. Figure 4 shows the age‐standardised incidence trends for brain and CNS tumours by sex and histology group. For men, the incidence rates for neuroepithelial tumours were highest across the whole time period, while for women, the rates for tumours of the meninges outperformed those for neuroepithelial tumours in the late 1980s and increased sharply, especially after the year 2000, with a peak in 2013 and a decrease thereafter (overall AAPC 2.4 (95% CI 1.7, 3.1); joinpoint in 2016 with APC (1980–2016) of 4.3 (3.8, 5.1) and APC (2016–2020) of −10.5 (−21.4, −4.2)). This is in line with the general increase in benign/borderline tumours in women after the year 2000 (Figure 1), as tumours of the meninges are mostly nonmalignant. In men, tumours of the meninges increased over the whole period with an AAPC of 1.3 (0.8, 2.1). An increase in sellar region tumours was observed in both sexes in more recent years.

### 3.2 Mortality

In 1980–1990, the overall age‐standardised mortality rate of malignant and benign/borderline brain and CNS tumours was 6.6 per 100,000 person‐years in men and 5.2 in women. In 2011–2021, the respective rates were 6.2 in men and 4.2 in women. Over the whole period (1980–2021), there was an AAPC of −0.3 (95% CI‐0.7, 0.1) for men and −0.7 (−1.0, −0.3) for women. Table 4 displays the age‐standardised mortality rates by sex, behaviour and period.

**TABLE 4:** Age‐standardised mortality rates of brain and CNS tumours (per 100,000 person‐years) by sex, behaviour and period, 1980–2021, canton of Zurich, Switzerland.

	Malignant tumours		Benign/borderline tumours	
	Men	Women	Men	Women
Period				
1980–1990	5.7	4.0	1.0	1.2
1991–2000	6.1	3.6	0.6	0.6
2001–2010	5.5	3.5	0.6	0.6
2011–2021	5.7	3.6	0.5	0.5

**FIGURE 5:**
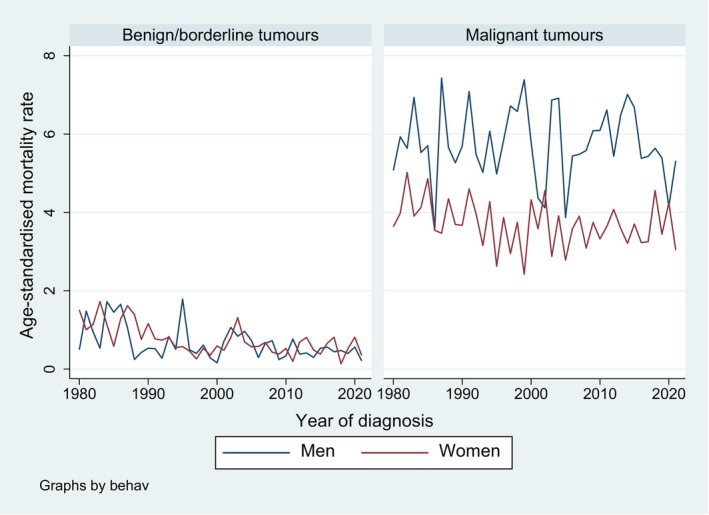
Trends in age‐standardised mortality rates for brain and CNS tumours (per 100,000 person‐years) from 1980 to 2021, by sex and behaviour, canton of Zurich, Switzerland.

Age‐standardised mortality rates were lower for benign/borderline than for malignant brain and CNS tumours among both sexes (Figure 5). While the mortality rates for benign/borderline tumours were similar in men and women, they were higher in men for malignant tumours. The trend was significantly decreasing for benign/borderline tumours (AAPC of −2.6 (−4.0, −1.2) for men and of −2.5 (−3.6, −1.4) for women) but stable for malignant tumours (AAPC of −0.1 (−0.5, 0.4) for men and of −0.3 (−0.7, 0.1) for women).

**FIGURE 6:**
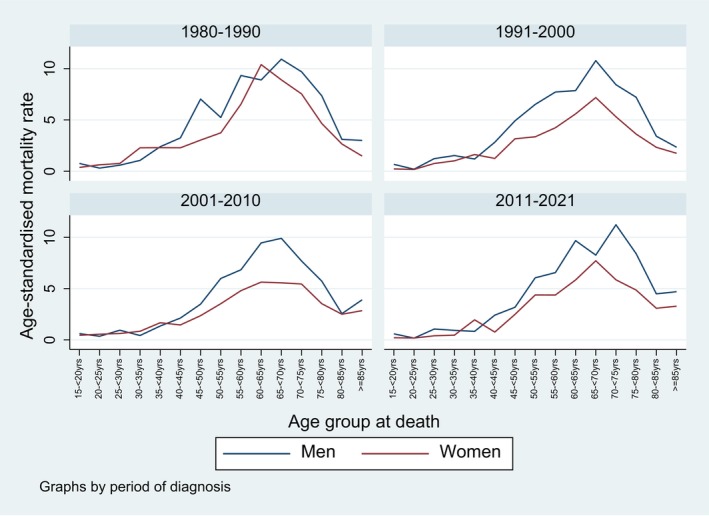
Age‐standardised mortality rates for brain and CNS tumours (per 100,000 person‐years) from 1980 to 2021, by sex, period and age group, canton of Zurich, Switzerland.

**FIGURE 7:**
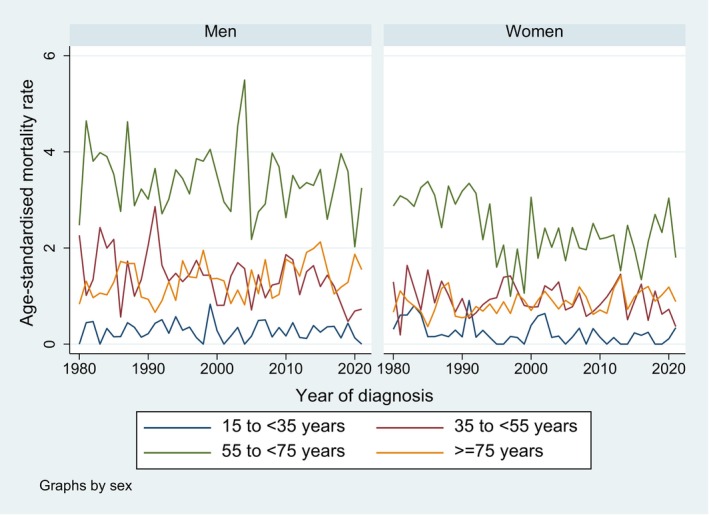
Trends in age‐standardised mortality rates for brain and CNS tumours (per 100,000 person‐years) from 1980 to 2021, by sex and age group, canton of Zurich, Switzerland.

Figure 6 shows the age‐standardised mortality rates across age groups, by period and sex. While there was no difference in mortality rates between men and women below age 40, men had slightly higher mortality rates in middle and older ages. There was no clear trend in mortality rates over time, also if stratified by age category (Figure 7). As for incidence, the highest mortality rates were observed in patients aged 55 to 74 years over the whole time period. The AAPC were significantly decreasing in 35–54 year old men (−1.4 [−2.4, −0.5]) and in 15–34 year old (−5.1 [−7.4, −4.0]) and in 55–74 year old women (−0.9 [−1.5, −0.4]), and significantly increasing in the 75+ age group in both sexes (men: 0.8 [0.1, 1.9], women: 0.7 [0.1, 1.6]). All other AAPC were nonsignificant.

### 3.3 Five‐Year Relative Survival

**FIGURE 8:**
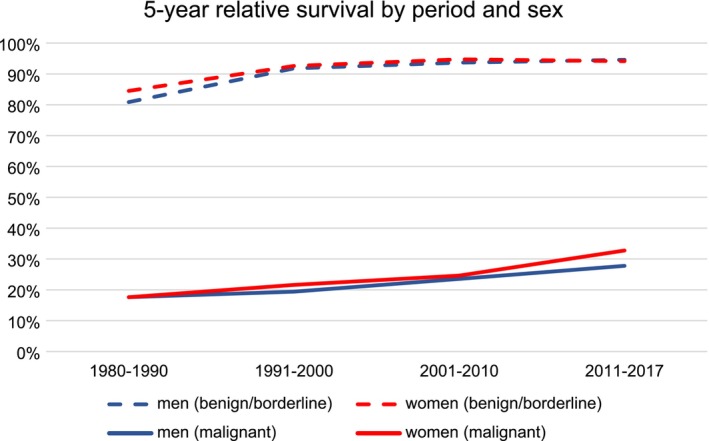
Trends in age‐standardised 5‐year relative survival for brain and CNS tumours from 1980 to 2021, by sex and behaviour, canton of Zurich, Switzerland.

Age‐standardised 5‐year relative survival for benign/borderline tumours increased from around 80% in 1980–1990 up to more than 90% in 2011–2017 (Figure 8). While it was slightly higher in women than in men during the first period, it was comparable in the later periods. For malignant tumours, 5‐year relative survival increased from below 20% in 1980–1990 to around 30% in 2011–2017 and was slightly higher in women, at least in some periods.

## 4 Discussion

We identified more than 10,000 incident cases of brain and CNS tumours in the canton of Zurich, Switzerland, between 1980 and 2021, of which about 40% were classified as malignant. More than 3500 deaths were counted in the same period, of which around 85% were classified as malignant. We observed sex differences in incidence rates, with higher rates for benign/borderline tumours in women and higher rates for malignant tumours in men. Furthermore, there was an increase in incidence rates over time for benign/borderline but not for malignant tumours. The latter increase was more pronounced in women than in men. As displayed in Figure 4, the increase in benign/borderline tumours in women was mainly driven by the large increase in tumours of the meninges (which were mostly classified as benign/borderline according to Table S2) starting after 2000. For both men and women, highest incidence rates were observed in the middle‐age groups between around 45 and 75 years.

Mortality rates were comparable for both sexes regarding benign/borderline tumours, while they were higher in men than women for malignant tumours. Five‐year relative survival increased slightly over time and was, as expected, clearly higher for benign/borderline compared with malignant tumours (more than 90% versus around 30% in the most recent period).

### 4.1 Comparison With Other Studies

A Swiss study including both benign/borderline and malignant brain and CNS tumours for the period 2010–2014 reported standardised incidence rates of 8.4 (men) and 5.1 (women) per 100,000 person‐years for malignant tumours and 6.6 (men) and 12.6 (women) for benign/borderline tumours [2]. While for malignant tumours, the rate for men was about 1.5 times higher compared with the rate for women, the opposite was observed for benign/borderline tumours with rates for women that were almost double the rates for men. This is in line with the results of the present study.

Global studies reported significant differences regarding the incidence of malignant brain tumours according to region: Between 2003 and 2007, the lowest rates were reported in Asian countries (ASR of around 2.5 to 3 per 100,000 person‐years) and the highest rates in Northern Europe and Canada (ASR of around 6.5) [5]. Based on Cancer Incidence in Five Continents, Volume X, covering also diagnoses between 2003 and 2007, rates of around 2.8 per 100,0000 person‐years were reported in Africa and up to 6.8 in Europe [4]. Furthermore, an analysis of the Global Burden of Disease 2019 study database revealed incidence rates for malignant brain tumours varying between 2 per 100,000 person‐years in the African region and almost 8 per 100,000 person‐years in the European region for men, and between 1.5 and almost 6 per 100.000 person‐years in women [15]. With around 7 to 8 per 100,000 person‐years for malignant tumours in men and 4 to 5 in women, our results fit well within the rates in other European regions and more generally in high‐income countries. Similar rates were also reported in New Zealand with 6.7 per 100,000 person‐years in males and 4.5 in females between 1995 and 2010 [16]. These observed differences between low‐ and high‐income countries may partly be explained by variations in data quality, such as underreporting of brain cancers in low‐income countries due to limited resources (both regarding diagnostic techniques and cancer registration), or overestimation of brain cancer due to potential misclassifications of brain metastases as primary brain tumours [15, 17]. Furthermore, an increased use of neuroradiological imaging techniques as diagnostic methods, specifically in high‐income countries, may have led to an increase in incidental findings of symptomless benign brain tumours that would not need any treatment [2].

Different genetic susceptibilities and environmental exposures have also been discussed as potential reasons for the global differences [17, 18]. Finally, differences in definitions and use of reporting standards may also play a role [19].

Even slightly higher than in our study, a CBTRUS study reported age‐standardised incidence rates of malignant brain and CNS tumours of 8.3 per 100,000 person‐years in men and 6.0 in women (overall 7.1 per 100,000 person‐years) in the USA between 2013 and 2017 [20]. Similar rates were observed in a Finnish study with 9.3 per 100,000 person‐years in men and 6.5 in women (overall 7.7 per 100,000 person‐years) [21] and in a Canadian study from 1992 to 2017 with average age‐standardised incidence rates of 8.4 per 100,000 person‐years in men and 6.5 in women (overall 7.9 per 100,000 person‐years) [22]. Males accounted for more than half (56.4%) of all diagnoses in the Canadian study, which is comparable to the 57.7% of malignant tumours that were diagnosed in men in Zurich and also to a Canadian study reporting 56% being diagnosed in men [23], but slightly higher compared to a global estimate from 2003 to 2007 of 54% being diagnosed in men [3].

Including both benign/borderline and malignant brain and CNS tumours, the CBTRUS study in the USA from 2016 to 2020 reported an age‐adjusted incidence rate of 24.8 per 100,000 person‐years (malignant: 6.9, non‐malignant: 17.9), with higher rates in females compared to males (27.8 versus 21.6 per 100,000 person‐years) [9]. This is slightly higher than what we found in Zurich (18.5 in men and 24.1 in women in the most recent period). While in the USA, 28% of the diagnosed tumours were malignant [9], this percentage was higher in Zurich at 39%.

We observed higher incidence rates for malignant brain and CNS tumours in men compared to women, but higher rates for benign/borderline tumours in women compared to men. Such a difference has also been reported in Italy [19] and the USA [9]. Sex differences with respect to incidence, mortality and survival have been observed in several cancers. Higher rates of gliomas are observed for men [24] whereas women have higher rates of meningiomas [25]. For meningiomas, there is evidence for tumour‐promoting effects of oestrogen and progesterone, but also for protective effects of testosterone [25]. On the other hand, oestrogen may have a protective effect on glioma development and growth, while exposure to androgens may increase the risk of glioma [24].

Increasing trends, specifically in benign/borderline tumours, have been reported in other countries as well. An English study reported very similar increases in CNS tumours from 13.0 per 100,000 person‐years in 1993 to 18.6 in 2017 with an AAPC of +1.5 (95% CI: 1.3, 1.7) [26]. While the ASR for malignant tumours remained stable, it increased for benign tumours with an AAPC of +2.6 (1.2, 4.0). According to the authors, this trend could be the result of the increasing use of neuroimaging, particularly CT head scans in individuals aged 65 years and older [26]. Similarly, an Italian study reported a stable incidence rate for malignant brain tumours between 1985 and 2005 but an increasing rate for benign brain tumours (APC of 6.2 [4.5, 7.9]) [19]. In the USA, a decrease was observed for malignant brain and CNS tumours for both males and females between 2007/08 and 2019, and an increase in nonmalignant brain and CNS tumours [9]. Increasing trends, specifically in nonmalignant tumours, may be attributable to improved diagnostic techniques [19], as no risk factors that may be responsible for the observed increase have been identified [27]. Furthermore, the number of CT scans has about doubled in Switzerland between 1998 and 2008 [28], which could also partly explain the increasing trend.

According to the Global Burden of Disease 2019 study database, mortality rates for malignant brain tumours varied between 1.8 per 100,000 person‐years in the African region and 5.4 in the European region for men, and between 1.3 and 3.5 in women [15], indicating that the rates for Europe are very similar to our results. Likewise, the CBTRUS analyses revealed a mortality rate for malignant brain and CNS tumours of 5.4 per 100,000 person‐years in men and 3.6 in women (overall 4.4 per 100,000 person‐years) [9].

The 5‐year relative survival rate for malignant brain and other CNS tumours was 36% in CBTRUS 2016–2020 [9], slightly higher than what we observed in Zurich. A study from Taiwan reported 5‐year relative survival rates of 27.5% (24.1%–30.9%) in 1997 and 32.8% (29.6%–35.9%) in 2012 [29] compared to survival rates in our study of 28% in men and 33% in women between 2011 and 2017. According to CONCORD‐3, 5‐year net survival of malignant brain tumours was in the range of 20%–40% in most countries in the years 2000–2014, with estimates for Switzerland for 2010–2014 of around 30% [8], which is in line with our estimates for Zurich for 2011–2017.

Not many studies have investigated relative survival for benign/borderline brain and CNS tumours. An Austrian study reported similar results for data from 2005 to 2010 (5‐year relative survival of around 96% compared to 94% in our study) [30]. According to CBTRUS, the 5‐year relative survival rate for nonmalignant brain and other CNS tumours was 91.8% [9], which is also comparable to our results.

The higher incidence in meningiomas that we observed in women compared to men and also the increasing trend was also observed in a CBTRUS study in the USA [31]. However, the increase in (non‐malignant) meningiomas in the USA was confined to the period 2005–2009, with a decrease from 2009 to 2015 in women and a stable rate in men [31]. In our study, the increase in women continued until around 2013, and the increase in men was observed over the whole period.

## 5 Strengths and Limitations

The strengths of this study were the long follow‐up period, the good data quality of the registry data including high completeness and almost complete follow‐up data, and the inclusion of both malignant and benign/borderline brain and CNS tumours. Furthermore, we stratified the analyses by sex, age group, behaviour and histological subtypes. However, the study also had some limitations. The analysis only included data from the canton of Zurich because registration in the other cantons of our registry started much later. Moreover, histological subtypes could not be analysed for mortality trends because no histology codes according to ICD‐O were available for death causes.

## 6 Conclusions

We observed an increase in incidence and a decrease in mortality rates for benign/borderline tumours, while both rates remained stable for malignant tumours between 1980 and 2021. The different trend demonstrates the importance of registering benign/borderline brain tumours in addition to malignant brain tumours in epidemiological, population‐based cancer registries. Five‐year relative survival improved over time, both for benign/borderline and for malignant tumours. The increasing incidence rates in benign/borderline tumours may be due to an increased use of improved diagnostic techniques, as reported in other countries. The improvement in relative survival may reflect earlier detection and better treatment options. Further technical developments therefore may result in future survival improvements.

## Author Contributions

**Miriam Wanner:** conceptualization, writing – original draft, methodology, formal analysis. **Flurina** **Suter:** writing – review and editing, formal analysis. **Manuela** **Limam:** writing – review and editing, data curation. **Dimitri** **Korol:** writing – review and editing, conceptualization, data curation, methodology. **Sabine** **Rohrmann:** conceptualization, writing – review and editing, supervision, methodology.

## Ethics Statement

In the Canton of Zurich, all cancer cases are registered with presumed consent and registered based on a decision from 1980 by the Zurich Government Council and the general registry approval from 1995 by the Federal Commission of Experts for professional secrecy in medical research. In this analysis, all data were used anonymously, and therefore, no approval was required from the Ethics Committee of the Canton of Zurich.

## Conflicts of Interest

The authors declare no conflicts of interest.

## Supporting information


**Table S1.** Definition of histopathological sub groups of brain and CNS tumours.

**Table S2.** Distribution of sub groups of brain and CNS tumours used for stratification, by behaviour, canton of Zurich, Switzerland, 1980 to 2021.

## Data Availability

The data that support the findings of this study are available on request from the corresponding author. The data are not publicly available due to privacy or ethical restrictions.
