# Supplementary material for: Incidence, Mortality and Survival Time Trends of Brain and CNS Tumours in the Canton of Zurich (Switzerland) Between 1980 and 2021
Source: Cancer Med. 2025 Jul 21;14(14):e71052. doi: 10.1002/cam4.71052 (PMC12277868; doi:10.1002/cam4.71052)
Supplement: Supplementary file 1 — Table S1. Definition of histopathological sub groups of brain and CNS tumours. [file CAM4-14-e71052-s001.docx]

**Supplementary Table 1**. Definition of histopathological sub groups of brain and CNS tumours

| **Sub group** | **Specific histopathology** | **ICD-O-3 histopathology codes** |  |
| --- | --- | --- | --- |
|  |  | malignant | non-malignant |
| Neuroepithelial tumors |  |  |  |
|  | Pilocytic astrocytoma | 9421/1, 9425/3 |  |
|  | Diffuse astrocytoma | 9381/3, 9400/3, 9410/3, 9411/3, 9420/3 |  |
|  | Anaplastic astrocytoma | 9401/3 |  |
|  | Glioblastoma | 9440/3, 9441/3, 9442/3, 9445/3 |  |
|  | Other astrocytomas | 9380/3, 9381/3, 9385/3, 9423/3, 9424/3, 9425/3 | 9384/1 |
|  | Oligodendroglioma | 9450/3 |  |
|  | Anaplastic oligodendroglioma | 9451/3 |  |
|  | Oligoastrocytoma | 9382/3 |  |
|  | Ependymoma | 9391/3, 9392/3, 9393/3 | 9383/1, 9394/1 |
|  | Neuronal - glial | 9390/3, 9505/3 | 8680/0, 8680/1, 8693/1, 9390/0, 9390/1, 9413/0, 9490/0, 9492/0, 9493/0, 9505/1, 9506/1, 9509/1 |
|  | Embryonal - medulloblastoma | 8963/3, 9470/3, 9471/3, 9473/3, 9475/3, 9477/3, 9500/3, 9508/3 |  |
| Tumors of cranial and spinal nerves | Nerve sheath tumors | 9540/3, 9560/3 | 9540/0, 9540/1, 9560/0, 9560/1 |
| Tumors of meninges |  |  |  |
|  | Meningioma | 9530/3, 9538/3, 9539/3 | 9530/0, 9530/1, 9531/0, 9532/0, 9533/0, 9534/0, 9535/0, 9537/0, 9538/1, 9539/1 |
|  | Mesenchymal – lipoma, haemangioma | 8800/3, 8801/3, 8815/3, 8910/3, 9150/3, 9370/3 | 8815/0, 8815/1, 9150/1, 9161/1 |
|  | Haemangioblastoma |  | 9161/1 |
| Lymphomas | Lymphoma | 9590/3, 9591/3, 9670/3, 9671/3, 9675/3, 9680/3, 9684/3, 9687/3, 9691/3, 9699/3, 9702/3 9714/3, 9715/3, 9727/3, 9728/3, 9751/3 |  |
| Germ cell tumours | Germinoma | 9060/3, 9064/3, 9081/3 | 9080/0 |
| Tumors of the sellar region |  |  |  |
|  | Tumors of the pituitary |  | 8270/0, 8271/0, 8272/0, 8280/0 |
|  | Craniopharyngioma |  | 9350/1, 9351/1, 9352/1 |
| Unclassified | Unclassified | 8000/3 | 8000/0, 8000/1 |

This table is based on: *Ostrom QT, Price M, Neff C, Cioffi G, Waite KA, Kruchko C, Barnholtz-Sloan JS. CBTRUS Statistical Report: Primary Brain and Other Central Nervous System Tumors Diagnosed in the United States in 2016-2020. Neuro Oncol. 2023;25(Supplement_4):iv1-iv99*.
