# Supplementary material for: Incidence, Mortality and Survival Time Trends of Brain and CNS Tumours in the Canton of Zurich (Switzerland) Between 1980 and 2021
Source: Cancer Med. 2025 Jul 21;14(14):e71052. doi: 10.1002/cam4.71052 (PMC12277868; doi:10.1002/cam4.71052)
Supplement: Supplementary file 2 — Table S2. Distribution of sub groups of brain and CNS tumours used for stratification, by behaviour, canton of Zurich, Switzerland, 1980 to 2021. [file CAM4-14-e71052-s002.docx]

**Supplementary Table 2**. Distribution of sub groups of brain and CNS tumours used for stratification, by behaviour, canton of Zurich, Switzerland, 1980 to 2021

| **Histology group** | **Behaviour** | | **Total** |
| --- | --- | --- | --- |
|  | **Malignant** | **Benign/borderline** |  |
| Neuroepithelial tumours | 3450 | 320 | 3770 |
| Tumours of the cranial/spinal nerves | 10 | 926 | 936 |
| Tumours of the meninges | 83 | 4272 | 4355 |
| Lymphomas | 262 | 0 | 262 |
| Germ cell tumours | 16 | 3 | 19 |
| Tumours of the sellar region | 1 | 570 | 571 |
| Unclassified/other tumours | 149 | 164 | 313 |
| **Total** | **3971** | **6255** | **10’226** |
